# Seroprevalence and Vaccination Determinants of Varicella Zoster Virus Among Pediatric and Adolescent Populations in Northern Lebanon

**DOI:** 10.3390/vaccines13111166

**Published:** 2025-11-15

**Authors:** Nourhan Farhat, Dima El Safadi, Jana Massoud, Sara Khalife

**Affiliations:** 1 Department of Medical Laboratory Technology, Faculty of Health Sciences, Beirut Arab University, Tripoli 11-5020, Lebanon; nourhanfarhat@hotmail.com (N.F.); jana.massoud2001@gmail.com (J.M.); 2 Department of Clinical Sciences, Liverpool School of Tropical Medicine, Liverpool L7 8XZ, UK

**Keywords:** varicella zoster virus, seroprevalence, vaccination, pediatric epidemiology, North Lebanon, public health, immunization policy

## Abstract

Background: Varicella zoster virus (VZV) remains a significant cause of pediatric morbidity in populations in Lebanon, yet comprehensive data on population immunity and vaccination uptake are limited. This study aimed to estimate VZV seroprevalence and identify factors associated with immunity and vaccine uptake among children and adolescents in Northern Lebanon. Methods: A cross-sectional study was conducted among 180 participants aged 1–18 years recruited from urban and rural settings in North Lebanon. After receiving informed parental consent, sociodemographic and clinical information were collected via structured questionnaires. Anti-VZV IgG and IgM antibodies were measured using validated Enzyme-Linked Immunosorbent Assays (ELISA). Associations with seropositivity and vaccination uptake were analyzed using multivariable logistic regression. Results: IgG seroprevalence was 79.4% (95% CI: 72.7–85.1), indicating prior exposure or immunization, while IgM antibodies, reflecting recent infection, were detected in 5.0% (95% CI: 2.3–9.4) of participants. Among vaccinated participants, IgG seropositivity was 63.6% (95% CI: 43.5–83.7) in the one-dose group and 89.5% (95% CI: 83.0–96.0) in the two-dose group. Completing the two-dose regimen was significantly associated with a higher IgG seropositivity (OR = 0.110, 95% CI: 3.2–52.4, *p* = 0.002). Parental reporting of history of varicella showed high sensitivity (99.0%) and overall accuracy (90.8%) in predicting seropositivity. Primary vaccination barriers included preference for natural infection (67%), perceived non-necessity (19%), and cost (10%). Regular pediatric follow-up strongly predicted vaccination (OR = 15.239, *p* < 0.001), whereas low parental awareness was associated with decreased vaccine uptake (OR = 0.027, *p* = 0.015). Conclusions: Suboptimal VZV vaccination coverage and persistent susceptibility underscore the need to integrate varicella vaccination into Lebanon’s national immunization schedule. Targeted educational efforts and enhanced pediatric healthcare engagement are critical to increasing vaccine uptake and reducing disease burden.

## 1. Introduction

Varicella zoster virus (VZV) is a strictly human, neurotropic alpha-herpesvirus responsible for causing varicella (chickenpox) upon primary infection, predominantly affecting children aged 1 to 9 years. The disease typically presents as a self-limiting febrile illness characterized by a pruritic vesicular rash that appears in successive crops over the trunk and face [1]. While usually benign in immunocompetent hosts, complications such as secondary bacterial infections, varicella pneumonia, and encephalitis can occur, particularly in immunocompromised individuals [2]. Following primary infection, VZV establishes lifelong latency in sensory dorsal root ganglia. Reactivation later in life can cause herpes zoster (shingles), a painful dermatomal vesicular rash that may be complicated by neurological issues such as Ramsay Hunt syndrome and postherpetic neuralgia [1,3].

Structurally, VZV is an enveloped virus with an icosahedral capsid encapsulating a linear double-stranded DNA genome encoding essential viral replication and latency proteins [4,5]. Transmission occurs primarily through respiratory droplets and direct contact with vesicular fluid, leading to initial replication in the respiratory mucosa, viremia, and dissemination to skin and sensory ganglia, where latency is established [4]. VZV is highly infectious, having an incubation period of approximately 14 days [6]. Clinical diagnosis primary relies on characteristic signs, but serological assays detecting anti-VZV IgM and IgG antibodies and molecular PCR tests are crucial, especially in atypical or complicated cases [7,8].

Globally, varicella remains a significant cause of morbidity. Before widespread vaccination, it caused millions of hospitalizations and thousands of deaths annually, disproportionately affecting children and immunocompromised populations [9]. The introduction of live-attenuated varicella vaccines, such as VARIVAX and the combined measles-mumps-rubella-varicella (MMRV) vaccine (ProQuad), has led to substantial reductions in disease incidence, hospitalizations, and mortality in high-income countries [10,11]. These vaccines are generally administered in two doses, beginning at 12–15 months of age with a booster at 4–6 years [6].

In the Middle East and North Africa (MENA) region, the epidemiology of VZV is heterogenous. Recent systematic reviews report seroprevalence of VZV-specific antibodies ranging from 22% to over 90%, influenced by factors such as age, climate, and vaccination policies [12]. For instance, population-based studies have found VZV IgG antibody seroprevalence of 94.6% in Iran, 84.1% in Turkey, 74.4% in Saudi Arabia, and 53.3% in Iraq, with higher seropositivity observed with increasing age [13,14,15,16]. Qatar was among the first in the region to implement a national varicella vaccination program, leading to improved vaccine coverage since 2002; however, disease incidence remains affected by high expatriate populations with variable immunity [17].

In Lebanon, varicella vaccination is currently excluded from the national immunization program and remains an optional, non-mandatory vaccine with low uptake (~21.4%), hindered largely by financial constraints and limited public health coverage [18]. Despite this, seroprevalence studies report high VZV IgG positivity rates—96.6% among school-aged adolescents and 93% among medical and paramedical university students—assessed by ELISA, indicating that immunity in these populations primarily results from natural infection rather than vaccination [19,20]. This paradox of high immunity amidst low vaccination uptake highlights the continued circulation of wild-type VZV and underscores vulnerabilities in current prevention strategies.

Accurate assessment of varicella-zoster virus (VZV) seroprevalence is crucial for understanding population immunity, guiding vaccination policies, and forecasting disease burden, including herpes zoster [17]. Serological surveys provide valuable insights into both vaccine-induced and infection-acquired immunity, the durability of protection, and the identification of subpopulations at greater risk who may benefit from targeted immunization efforts [21]. Despite this importance, data on VZV seroepidemiology and vaccination determinants among pediatric and adolescent populations in Northern Lebanon remain scarce. Previous studies have identified several determinants of varicella immunity and vaccination uptake, including age, household size, parental education, vaccine awareness, and engagement with pediatric healthcare services [12]. Globally, additional factors such as vaccine hesitancy, healthcare system infrastructure, and provider recommendations have also been recognized as key influences on vaccination behavior [21].

Given these gaps in regional data and the multifactorial nature of varicella immunity, the present study aimed to assess the seroprevalence of VZV-specific antibodies among children and adolescents in Northern Lebanon and to examine how vaccination status, sociodemographic factors, and healthcare engagement influence immunity levels. We further sought to identify barriers and determinants of vaccine uptake to inform public health strategies and optimize immunization coverage in Lebanon and similar settings.

## 2. Materials and Methods

### 2.1. Study Design

A cross-sectional, community-based study was conducted among the pediatric population of North Lebanon (34.4381° N, 35.8308° E) between June and July 2024. The study included 180 children and adolescents aged 1 to 18 years, recruited from both urban and rural settings using a convenience sampling strategy. Although convenience sampling was used, recruitment was diversified across multiple urban and rural sites to enhance geographic and demographic representation. Exclusion criteria comprised any child with a history of immunocompromised state, chronic illnesses, or recent blood transfusion, to ensure serological test validity.

### 2.2. Data and Sample Collection

Upon obtaining written informed consent, parents or legal guardians completed a structured, questionnaire designed to collect comprehensive information. This included demographic data (age, gender, residence), household characteristics (parental education level, number of siblings), vaccination history (number of doses and age at administration), reasons for non-vaccination, history of pediatric and medical follow-up, prior clinical varicella infection, awareness and perceptions regarding varicella-zoster virus (VZV), and any previous VZV-related laboratory testing.

Venous blood samples (3–4 mL) were collected from each participant by trained phlebotomists using strict aseptic techniques. Samples were immediately transported in iceboxes to the Biomedical Laboratory at Beirut Arab University (Tripoli campus), where they were centrifuged for serum separation and subsequently stored at –20 °C until serological analysis was performed.

The standardized procedure for sample collection, combined with the detailed and validated questionnaire, ensured reliable data collection adequately suited to address the study’s objectives of estimating seroprevalence and identifying vaccination determinants.

### 2.3. Serological Testing

Serum samples were tested for anti-VZV IgM and IgG antibodies using EUROIMMUN ELISA kits (EUROIMMUN Medizinische Labordiagnostika AG, Lübeck, Germany), following the manufacturer’s instructions. The IgM and IgG assays demonstrated a sensitivity and specificity of 100% per the manufacturer’s data and literature validation. Results were interpreted according to EUROIMMUN^®^ criteria as positive (≥11 IU/mL), borderline (≥8 and <11 IU/mL), or negative (<8 IU/mL). Absorbance was measured using a HumaReader HM spectrophotometer (Human GmbH, Wiesbaden, Germany) with each ELISA run including both positive and negative controls. All measurements were performed in duplicate, and intra- and inter-assay coefficients of variation were maintained below 10%.

Serological classification using the defined EUROIMMUN thresholds guided all subsequent analyses. Samples yielding borderline results were considered indeterminate and excluded from multivariate regression modeling to ensure clear categorization of immunological status. This threshold-based approach provided a robust analytic framework for estimating seroprevalence and evaluating predictors of VZV immunity and vaccine uptake.

### 2.4. Statistical Analysis

Data were analyzed using IBM SPSS Statistics for Windows, version 24.0 (IBM Corp., Armonk, NY, USA). Variables were classified according to their measurement scale to guide appropriate statistical methods: categorical variables such as gender, residence, and vaccination status were treated as nominal; ordinal variables such as parental education level and awareness were categorized into ranked groups; continuous variables including age and number of siblings were treated as ratio scale. Categorical variables were described as frequencies and percentages, while continuous variables as means and SDs. Student’s t-test was used for continuous variables, and chi-square tests or Fisher’s exact test for categorical variables. Logistic regression models identified independent predictors of seropositivity, presenting odds ratios (OR) and 95% confidence intervals (CI). An “Indeterminate” category was assigned for missing data (participants with an indeterminate vaccination status (*n* = 20, 11.1%) were excluded from the logistic regression analysis to ensure accurate estimation of factors associated with vaccination uptake). Statistical significance was set at a *p*-value threshold of <0.05 (two-tailed). For assessment of parental reporting validity, serology was the reference standard, with sensitivity, specificity, positive predictive value, negative predictive value, and overall accuracy calculated per standard formulas [20].

### 2.5. Ethical Statement

The study was initiated after approval by the institutional review board (IRB) of Beirut Arab University (IRB: 2024-H-0189-HS-M-0618). Written parental consent was required for each participant.

## 3. Results

### 3.1. Participant Characteristics

The study involved 180 children and adolescents, aged 1–18 years, comprising 41.7% males (*n* = 75) and 58.3% females (*n* = 105). Age distribution was 0–6 years (2.8%, *n* = 5), 7–12 years (47.8%, *n* = 86), and 13–18 years (49.4%, *n* = 89). Urban residents accounted for 61.1% (*n* = 110) with 38.9% (*n* = 70) living in rural areas. Fathers’ educational levels were primary (20.6%, *n* = 37), complementary-secondary (46.7%, *n* = 84), and university degree (32.8%, *n* = 59), while mothers’ educational attainment showed 33.3% (*n* = 60) primary, 48.3% (*n* = 87) complementary-secondary, and 18.3% (*n* = 33) university degree. The mean (SD) number of siblings per participant was 3.36 (± 1.48). Vaccination against varicella was reported by 60% (*n* = 108), among whom 79.6% (*n* = 86) had completed the two-dose schedule. Regular pediatric follow-up was reported for 79.4% (*n* = 143) of participants, while 63.9% (*n* = 115) reported a prior history of varicella infection. Parental awareness of varicella and shingles was high in 18.9% (*n* = 34), moderate in 76.7% (*n* = 138), and absent in 4.4% (*n* = 8). Additionally, 33.3% (*n* = 60) of parents perceived varicella and shingles as serious infections (Table 1).

### 3.2. VZV Seroprevalence

Overall, 79.4% (143/180; 95% CI: 72.7–85.1) of participants were seropositive for anti-VZV IgG antibodies, indicating immunity. Notably, participants seropositive IgG had a significantly higher mean number of siblings (3.78 ± 1.47) compared to seronegative participants (3.24 ± 1.46; *p* = 0.049) (Table 1). However, in the multivariate logistic regression, the number of siblings was not an independent predictor of VZV IgG seropositivity (*p* = 0.296) (Table 2). Recent infection indicated by IgM seropositivity was found in 5.0% (9/180; 95% CI: 2.3–9.4) with no significant associations with demographic or clinical factors (Table 1).

### 3.3. Vaccination and Immunity

IgG seropositivity varied significantly according to the number of varicella vaccine doses received (*p* = 0.006). To further examine this relationship, participants were stratified by vaccination dose. Seropositivity was observed in 63.6% of individuals who received one dose (14/22) and in 89.5% of those who received two doses (77/86) (Table 1). These findings indicate a higher level of serological protection among participants who completed the full two-dose regimen compared with those who received only a single dose. Multivariate logistic regression analysis further confirmed that receiving two vaccine doses was significantly associated with higher IgG seropositivity (OR = 0.110; 95% CI: 3.2–52.4; *p* = 0.002) (Table 2).

Among participants whose parents reported a history of varicella infection, 72.0% were IgG-positive, indicative of immunity, compared to only 28.0% IgG positivity among those without a parental history of varicella (*p* < 0.001) (Table 1). Using seropositivity as the reference standard, parental reporting of varicella infection demonstrated high diagnostic accuracy with sensitivity of 99.0% (95% CI: 95.6–99.9), specificity of 67.5% (95% CI: 53.0–79.7), positive predictive value (PPV) of 89.5% (95% CI: 83.4–93.8), a negative predictive value (NPV) of 96.1% (95% CI: 85.1–99.3) and an overall accuracy of 90.8%.

### 3.4. Reasons for Non-Vaccination

**Figure 1:**
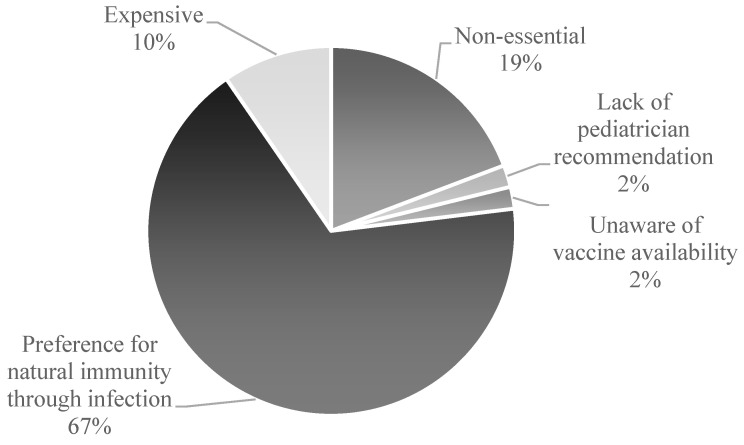
Parental Reasons for Vaccine Noncompliance in Childhood Immunization.

Among unvaccinated participants (*n* = 52), the predominant reason reported was a parental preference for natural infection to confer immunity (67%, *n* = 35). Other cited barriers included perceived lack of necessity (19%, *n* = 10), financial constraints (10%, *n* = 5), lack of awareness of the vaccine (2%, *n* = 1), and absence of pediatrician recommendation (2%, *n* = 1) (Figure 1).

### 3.5. Predictors of Vaccination Uptake

Multivariate logistic regression analysis of participants with definite vaccination status revealed that regular pediatrician follow-up was the strongest positive predictor of vaccination uptake (OR = 15.239; 95% CI: 5.0–45.8; *p* < 0.001). Conversely, parental lack of awareness about VZV significantly reduced the likelihood of vaccinating (OR = 0.027; 95% CI: 0.0–0.4; *p* = 0.015), compared to parents reporting high awareness (Table 3).

## 4. Discussion

This study evaluated the seroprevalence of IgM and IgG anti-VZV among children and adolescents in Northern Lebanon, reporting an overall IgG seropositivity of 79.4%. This prevalence exceeds that reported in some regional studies, such as 53.3% in Iraq [16] and 68% in Saudi Arabia [22]. However, it remains lower than figures from the United States, where IgG seropositivity reaches approximately 86% among children aged 6–11 years and ≥99% in adults [23]. Comparable data from Lebanon indicate seroprevalence exceeding 90% among adolescents and young adults, primarily reflecting natural infection in the context of absent routine varicella vaccination [17,20].

Consistent with previous Lebanese findings, no significant associations were observed between IgG serostatus and age, gender, geographic district, parental occupation or education [20]. The earlier reported relationship between smaller family size and seronegativity was not significant after multivariate adjustment, which may reflect differences in sample size, population demographics, or other confounding factors. These non-significant findings suggest that the influence of such sociodemographic attributes on varicella immunity may be more subtle or context-dependent than anticipated. It is also possible that their effects are overshadowed by stronger determinants, such as vaccination status and engagement with pediatric healthcare services.

Our findings underscore the critical importance of completing the recommended two-dose varicella vaccination schedule. Single-dose recipients exhibited significantly lower seropositivity, aligning with evidence from Spain and the United States that two doses confer greater and more durable immunogenicity [24]. Notably, population-level breakthrough outbreaks under one-dose programs led to US policy revisions mandating two doses, which subsequently decreased incidence and hospitalizations [2]. The two-dose varicella vaccination regimen has been shown to provide durable immunity, with studies reporting long-term protection extending at least 10 years post-vaccination. The incidence of breakthrough varicella was significantly lower among individuals who received two doses, and the risk of breakthrough infections did not appear to increase over time in this group [6]. Current evidence suggests that booster doses beyond the standard two are unnecessary [25].

While household size initially correlated with seropositivity, vaccination status predominates as the determinant of sustained immunity, consistent with a previous study [26]. Parental recall of varicella infection was highly sensitive though moderately specific, reaffirming its utility in estimating immunity within epidemiological contexts [26]. Parental awareness strongly influenced vaccination uptake, with unawareness significantly diminishing vaccination likelihood (OR = 0.027, *p* = 0.015). This mirrors findings demonstrating positive correlations between knowledge and vaccine acceptance [27]. Our findings indicate a vaccination coverage of 60.0% among study participants, substantially higher than the previously reported national uptake of approximately 21.4% for the varicella vaccine in Lebanon [18]. This discrepancy likely reflects selection bias inherent in our convenience sampling strategy. Although recruitment was diversified across multiple urban and rural sites to enhance geographic and demographic representation, the sample may still over represent families with greater access to healthcare services and higher vaccination awareness. This potential bias should be considered when interpreting our findings. The reasons for non-vaccination observed in this cohort may not fully reflect those in the broader population, where limited healthcare access, socioeconomic constraints, and lower vaccine awareness may play a larger role. Therefore, our results are most representative of families with relatively higher healthcare access and vaccination awareness and should be interpreted within this context [28].

Vaccine hesitancy, defined as the reluctance or refusal to vaccinate despite vaccine availability, remains a significant impediment to optimal varicella immunization coverage worldwide. A recent meta-analysis including data from over 30 countries estimated that 21.1% of parents routinely express vaccine hesitancy [29]. Sociocultural context critically shapes vaccine attitudes; for example, in the United States, rural communities frequently exhibit lower vaccination coverage due partly to limited healthcare access [30]. In the Middle East, deeply rooted cultural beliefs, misinformation, and limited vaccine accessibility contribute to persistent hesitancy, hindering consistent implementation of universal varicella vaccination (UVV) programs despite rising disease incidence and morbidity [12]. Vaccine hesitancy was notably driven by preference for natural immunity, cost concerns, and perceived vaccine necessity—barriers well documented in global and regional contexts [29]. Public health messaging must address misconceptions, particularly dispelling beliefs that natural infection is safer or preferable, by emphasizing vaccination’s role in preventing serious complications including herpes zoster [31].

Pediatrician engagement markedly increased vaccination rates (OR = 15.239, *p* < 0.001), underscoring healthcare providers’ pivotal role in parental decision-making. Evidence shows that effective provider communication and education substantially elevate immunization coverage [32], highlighting a strategic avenue to enhance vaccine uptake.

The 5.0% IgM seroprevalence indicates low recent infection rates, consistent with partial population immunity. Comparable seroprevalence levels have been reported in neighboring Syria and Israel, reflecting progressive population immunity accumulation through infection and evolving vaccination efforts [17,33].

School-based vaccination programs offer effective platform to increase coverage, enhance equity, and reduce disease burden among underserved populations [34,35]. For instance, a recent study reported a 5.5% increase in varicella vaccination coverage following school-based interventions [36], supporting their consideration in Lebanon’s immunization strategy. Given Lebanon’s private-sector-only varicella vaccination and lack of national program inclusion [37], integrating vaccination into school health initiatives represents a feasible and impactful strategy, as demonstrated elsewhere in the region [38].

Future research employing longitudinal designs should evaluate vaccine-induced immunity durability and behavioral determinants of vaccine acceptance. Strengthening provider training and tailoring education to community needs remain priorities to overcome hesitancy and improve varicella control.

This study provides valuable regional epidemiological data by determining varicella immunity and identifying key behavioral factors influencing vaccination uptake in Northern Lebanon, a population and setting with limited prior surveillance. Our findings provide a scientific basis to inform local immunization policies, particularly regarding the implementation of two-dose vaccination regimens and targeted educational interventions. This work thus fills a critical knowledge gap and serves as a foundation for future research and public health strategies aimed at enhancing varicella control in Lebanon and broader Middle Eastern contexts.

### Limitations

This study’s findings derive from a cross-sectional sample in North Lebanon, and may not be generalizable nationally due to regional population heterogeneity. The cross-sectional design inherently limits causal inference, as data were collected at a single point in time, preventing the establishment of temporal relationships between exposures and outcomes. The use of a convenience sampling approach, despite recruitment across urban and rural sites, may have introduced selection bias, as evidenced by the observed vaccination coverage (60.0%) exceeding the national estimate (21.4%), suggesting greater healthcare access and awareness among participants. The relatively small sample size (*n* = 180) and the low representation of children aged 0–6 years (2.8%, *n* = 5) may have limited statistical power for detecting subtle or subgroup-specific effects and reduced the precision of estimates for key vaccination groups. Reliance on parental recall for vaccination and infection histories introduces potential recall bias and misclassification, which may affect the accuracy of reported associations. Future studies employing larger, nationally representative samples and prospective designs, supported by verification of vaccination histories through medical records or immunization registries, are warranted to validate and expand upon these findings.

## 5. Conclusions

This study provides valuable insights into VZV seroprevalence and vaccination determinants among children and adolescents in Northern Lebanon. High immunity rates and the critical role of complete two-dose vaccination highlight the need for reinforcing varicella immunization. Parental awareness and pediatrician engagement emerged as pivotal factors influencing vaccine uptake. Implementing varicella vaccination within school-based public health programs represents a strategic opportunity to reduce VZV disease burden nationally and warrants urgent policy consideration.

## Figures and Tables

**Table 1:** Characteristics of participants, and serological profiles for IgM and IgG anti-VZV antibodies.

Variable	Total Count *n* (%)	Positive Anti-VZV IgM *n* (%)	Negative Anti-VZV IgM *n* (%)	*p*	Positive Anti-VZV IgG *n* (%)	Negative Anti-VZV IgG *n* (%)	*p*
ELISA Results	180	9 (5.0%)	171 (95.0%)	-	143 (79.4%)	37 (20.6%)	-
Gender				0.493			0.180
Male	75 (41.7%)	5 (55.6%)	70 (40.9%)		56 (39.2%)	19 (51.4%)	
Female	105 (58.3%)	4 (44.4%)	101 (59.1%)		87 (60.8%)	18 (48.6%)	
Age groups (years)				0.802			0.993
0–6	5 (2.8%)	0 (0.0%)	5 (2.9%)		4 (2.8%)	1 (2.7%)	
7–12	86 (47.8%)	5 (55.6%)	81 (47.4%)		68 (47.6%)	18 (48.6%)	
13–18	89 (49.4%)	4 (44.4%)	85 (49.7%)		71 (49.7%)	18 (48.6%)	
Area of residence				0.486			0.817
Urban	110 (61.1%)	7 (77.8%)	103 (60.2%)		88 (61.5%)	22 (59.5%)	
Rural	70 (38.9%)	2 (22.2%)	68 (39.8%)		55 (38.5%)	15 (40.5%)	
Father’s educational level				0.398			0.795
Primary education	37 (20.6%)	2 (22.2%)	35 (20.5%)		28 (19.6%)	9 (24.3%)	
Complementary-secondary	84 (46.7%)	6 (66.7%)	78 (45.6%)		67 (46.9%)	17 (45.9%)	
University	59 (32.8%)	1 (11.1%)	58 (33.9%)		48 (33.6%)	11 (29.7%)	
Mother’s educational level				0.281			0.508
Primary education	60 (33.3%)	5 (55.6%)	55 (32.2%)		50 (35.0%)	10 (27.0%)	
Complementary-secondary	87 (48.3%)	4 (44.4%)	83 (48.5%)		66 (46.2%)	21 (56.8%)	
University	33 (18.3%)	0 (0.0%)	33 (19.3%)		27 (18.9%)	6 (16.2%)	
Number of siblings				0.462			
Mean (SD)	3.36 (1.482)	3.00 (1.50)	3.37 (1.48)		3.78 (1.47)	3.24 (1.46)	0.049 *
Vaccination status				0.530			0.088
Yes	108 (60.0%)	4 (44.4%)	104 (60.8%)		91 (63.6%)	17 (45.9%)	
No	52 (28.9%)	4 (44.4%)	48 (28.1%)		36 (25.2%)	16 (43.2%)	
Indeterminate	20 (11.1%)	1 (11.1%)	19 (11.1%)		16 (11.2%)	4 (10.8%)	
-If yes: (a) The number of doses received				0.815			0.006 *
1	22 (20.4%)	1 (25.0%)	21 (20.2%)		14 (15.4%)	8 (47.1%)	
2	86 (79.6%)	3 (75.0%)	83 (79.8%)		77 (84.6%)	9 (52.9%)	
(b) Age at first and subsequent vaccine dose				0.800			0.092
Indeterminate	16 (14.8%)	0 (0.0%)	16 (15.4%)		14 (15.4%)	2 (11.8%)	
One single dose received at 1 y.o.	21 (19.4%)	1 (25.0%)	20 (19.2%)		14 (15.4%)	7 (41.2%)	
Two doses received: First at 1 y.o., second at non recommended age	5 (4.7%)	0 (0.0%)	5 (4.8%)		4 (4.4%)	1 (5.9%)	
First dose at 1 y.o. and second dose at the recommended age [4–6 y.o.]	66 (61.1%)	3 (75.0%)	63 (60.6%)		59 (64.8%)	7 (41.2%)	
Pediatrician follow-up				0.899			0.274
Yes	143 (79.4%)	7 (77.8%)	136 (79.5%)		116 (81.1%)	27 (73.0%)	
No	37 (20.6%)	2 (22.2%)	35 (20.5%)		27 (18.9%)	10 (27.0%)	
History of varicella				0.492			<0.001 *
Yes	115 (63.9%)	7 (77.8%)	108 (63.2%)		103 (72.0%)	12 (32.4%)	
No	65 (36.2%)	2 (22.2%)	63 (36.8%)		40 (28.0%)	25 (67.6%)	
Parents’ awareness of varicella and shingles				0.380			0.170
Very aware	34 (18.9%)	2 (22.2%)	32 (18.7%)		31 (21.7%)	3 (8.1%)	
Somewhat aware	138 (76.7%)	6 (66.7%)	132 (77.2%)		106 (74.1%)	32 (86.5%)	
Not aware	8 (4.4%)	1 (11.1%)	7 (4.1%)		6 (4.2%)	2 (5.4%)	
Parents’ perception of varicella and shingles severity				1.000			0.192
Yes	60 (33.3%)	3 (33.3%)	57 (33.3%)		51 (35.7%)	9 (24.3%)	
No	120 (66.7%)	6 (66.7%)	114 (66.7%)		92 (64.3%)	28 (75.7%)	

*: Significant, y.o.: year old.

**Table 2:** Multivariate analysis of factors associated with IgG anti-VZV positivity.

Variable	Positive IgG Anti-VZV Antibodies *n* (%)	Negative IgG Anti-VZV Antibodies *n* (%)	Odds Ratio	95% CI	*p*
Number of siblings	3.78 (1.47)	3.24 (1.46)	1.280	(0.8–2.0)	0.296
Number of doses received					
1 dose	14 (15.4%)	8 (47.1%)	0.110	(3.2–52.4)	0.002 *
2 doses (reference)	77 (84.6%)	9 (52.9%)	-	-	-

*: Significant.

**Table 3:** Multivariate analysis of factors associated with parental decision on Varicella Vaccination.

Variable	Odds Ratio	95% CI	*p*
Father’s Educational Level			
Primary education (Reference)	0.648	(0.1–2.3)	0.532
Complementary-Secondary	0.810	(0.2–2.4)	0.705
University	-	-	-
Mother’s Educational Level			0.161
Primary education (Reference)	0.768	(0.1–3.0)	0.713
Complementary-Secondary	1.925	(0.5–7.2)	0.327
University	-	-	-
Pediatrician Follow-Up			
Yes	15.239	(5.0–45.8)	<0.001 *
No (Reference)	-	-	-
Parents Familiarity with Varicella and Shingles			
Very Familiar (Reference)	-	-	-
Somewhat Familiar	0.246	(0.0–1.3)	0.129
Not Familiar	0.027	(0.0–0.4)	0.015 *
Parents Perception of Varicella and Shingles as Serious			
Yes	1.731	(0.5–5.5)	0.359
No (Reference)	-	-	-

*: Significant.

## Data Availability

Dataset available on request from the authors.

## Abbreviations

The following abbreviations are used in this manuscript:

VZV	Varicella zoster virus
ELISA	Enzyme-Linked Immunosorbent Assays
MMRV	measles–mumps–rubella–varicella
MENA	Middle East and North Africa
